# Using Artificial Intelligence-Based Collaborative Teaching in Media Learning

**DOI:** 10.3389/fpsyg.2021.713943

**Published:** 2021-10-13

**Authors:** Weijun Wang, Zhenhuan Liu

**Affiliations:** ^1^School of Creative Design, Shanghai Institute of Commerce & Foreign Languages, Shanghai, China; ^2^Department of Computer Engineering, Dongguan Polytechnic, Dongguan, China

**Keywords:** artificial intelligence, collaborative teaching, digital media education, teaching innovation, learning effectiveness

## Abstract

With the advent of the 5G era, humans must not only learn the knowledge and skills of cross-border integration but must also get to grips with the breadth and efficiency of artificial intelligence (AI) technology in order to jointly overcome current difficulties and create a happy and beautiful life. In this article, we use the example of an elementary school to discuss the decision-making factors that influence teachers when choosing AI technology, where the digital content of schools is imported into artificial intelligence-based collaborative teaching. After discussing the relevant literature, this study will introduce the concept of digital media education, and then compare the development and application of smart technology and human-computer collaborative teaching methods, describing three key aspects and factors that influence elementary school teachers’ choice of AI technology. There are 12 evaluation criteria in total. After the completion of expert questionnaires and data analysis, it was found that the main factors affecting teachers’ choice of AI technology are “collaborative tasks,” “functional characteristics,” and “modeling characteristics.” In terms of evaluation criteria, the four most important aspects were found to be “learning assistance,” “security,” “teaching observation,” and “record review.” The results of this research analysis will help provide a reference for digital content development and individual recommendation services. In future work, this study can further discuss teaching innovations in digital media education, aimed at improving the quality and effectiveness of teaching and learning.

## Introduction

In 2020, the COVID-19 pandemic became a global emergency, and the way in which interpersonal interaction was conducted changed dramatically. The trend of technology-assisted life is becoming more and more obvious. Teachers have begun to teach online, and students, too, have become familiar with this mode of education; governments have also made use of online platforms to help the public and refute rumors. In line with the trends of the digital economy and 5G era, the digital content industry has introduced artificial intelligence (AI) for cross-domain integrated innovative applications, and it has created new platform services and the experience economy. This is a new trend in the development of AI technology education for the future (Anita and Judit, 2016; Zhu, 2020).

The line between humans and machines is becoming more and more blurred with the development of artificial intelligence and biotechnology. Therefore, people must continually stay abreast of new technology and improve their abilities in order to adapt to contemporary life, finding meaning in and learning to value their existence. In the future, this study hopes to use AI technology to construct ubiquitous learning; to provide fair and inclusive educational opportunities; and to promote personalized learning and improve learning effectiveness. Therefore, education units must not only adjust their teaching and learning methods but also train students to adapt to modes of learning in the AI era. The nature of work literacy and work-related skills is changing at a dramatic rate, with the implementation of dual-teacher collaborative teaching, the introduction of education management information systems, formulation of public policies, and application of research in the field of AI education. The era of Industry 4.0 has seen robots replacing traditional labor. AI courses have been incorporated into basic education curriculum planning. AI textbooks and teaching plan examples for high schools, middle schools, and elementary schools have been completed and launched, and relevant promotional studies continue to be provided to teachers below high school (Omar et al., 2013; Kandlhofer et al., 2016).

Elementary school teachers formed the research group for this study, and this study explored the factors affecting decision-making in relation to digital content, where artificial intelligence was imported into collaborative teaching. Data were collected through questionnaire surveys, and the analytic hierarchy process (AHP) hierarchical analysis method was used to enable us to understand (and model) the decision-making characteristics of elementary school teachers in terms of AI technology and to determine the weighting of the relationship between perception of related factors (such as functional characteristics and collaborative tasks). This study then summarized and sorted the decision-making factors influencing elementary school teachers’ choice of AI technology-based collaborative teaching, as a reference for digital content development and individual recommendation services. The research objectives are summarized as to ascertain whether, after the introduction of digital content into artificial intelligence-based collaborative teaching, teachers attach importance to the shape, function, and role of AI technology. To determine the relative weight of decision-making factors associated with elementary school teachers’ selection of AI technology for the introduction of digital content into artificial intelligence-based collaborative teaching, this research mainly focused on all teachers in elementary schools. The research subject consisted of teachers, including qualified full-time teachers, acting teachers, hourly acting teachers, directors, and principals. The term “teachers” refers to teachers teaching in the school during the school year, but it does not include office workers, nurses, general workers, kitchen workers, or contracted personnel (Liu et al., 2014; Salavati et al., 2016; Chen et al., 2021a).

## Literature Review

### Intelligent Education

Innovative teaching applications emphasize deep-learning methods (such as topic inquiry, peer cooperation, flipped autonomy, and task-oriented or problem-oriented learning) or multiple learning modes (such as inter-school, international, and distance learning) and the possibilities for cultivating future students across the board. Key basic capabilities include globalization, cooperation, and innovation.

The government has allocated funds for the improvement of campus technology environments (i.e., establishing a smart network environment; building interactive software and hardware; introducing IoT sensors into classrooms; and purchasing online learning and mobile learning resources, etc.) in order to create a seamless learning experience, one where students are eager to learn and exhibit behaviors for learning at any time in different contexts (including those where equipment or vehicles are moved to help virtual IoT technology to try and change the personalized service provided by school teachers where students quickly switch between different situations and achieve the effect of learning transfer; Zhang et al., 2012; Chen et al., 2019a; 2021b). Therefore, by using cloud teaching and teachers, students, and staff, a “smarter” campus can utilize the interactive integration mode of campus resources to integrate a school’s curriculum teaching, administrative management, and learning. System and campus resources are integrated with the application system to improve the quality of teaching and then realize the learning mode of intelligent services and management (Mautone and Mayer, 2001). Smart campuses have become the focus of education reform in advanced countries around the world. Governments in Asia, Europe, and the United States have formulated policies to promote smart campus innovation and integration services and have compiled huge budgets to actively attack the international market (Chevalier et al., 2016; Suhaebar and Irokatun, 2019).

### Hierarchical Structure Analysis Method

The AHP is mainly used in uncertain situations and decision-making problems with several evaluation criteria. The AHP builds a hierarchy of factors in complex relationships, enabling comparison between pairs of possible factors in terms of importance. AHP theory is simple and practical. It also refers to the opinions of many experts. Since its development, it has been widely adopted by research institutions in various countries. Its application range is quite wide, especially in planning, forecasting, judgements, resource allocation, and investment portfolio trial calculations. Both have good results. The basic division of the evaluation scale of the hierarchical analysis method includes five items, namely “equally important,” “slightly important,” “quite important,” “extremely important,” and “absolutely important.” Values of 1, 3, 5, 7, or 9 can be assigned; the other four are between the five basic scales, and a value of 2, 4, 6, or 8 can be assigned (Anita and Judit, 2016; Zi-yun and Sung-chiang, 2019).

#### Operational Steps in Hierarchical Analysis

When the AHP has to deal with complex issues (such as multi-objective or multi-criteria decision-making evaluations), it mainly includes these steps:

Step 1. Define the problem.
Step 2. Establish a hierarchical structure.
Step 3. Design the questionnaire and survey.
Step 4. Establish a pairwise comparison matrix.

A paired comparison matrix is established to measure the results of the comparison between two elements of the questionnaire. When comparing the results of *n* factors in pairs, the upper triangular part of the comparison matrix is held to be A, and values in the lower triangular part represent the reciprocal of the relative position value of the upper triangular part, forming a paired comparison matrix form (Song et al., 2011; Salavati et al., 2016; Huang et al., 2019a).

Step 5. Calculate the eigenvector and the maximum eigenvalue.

Because the pairwise comparisons reflect the judgements of decision-makers, subjective judgements will differ from the actual values. There must be a certain degree of difference, which is inevitable and cannot be avoided; *n* is therefore replaced by the largest eigenvalue in the matrix.

Step 6. Verify the consistency.

The consistency index (C.I.) and the consistency ratio (C.R.) are used to verify the consistency of the paired comparison matrix (Grandzol, 2005; Arroyo et al., 2015; Munier, 2021).

##### Consistency Index

The consistency index obtained by the eigenvector method and the difference between *n* (matrix dimension) and the calculation can be used as a benchmark for judging the degree of consistency.

##### Consistency Ratio

The consistency index generated from the up-and-down value matrix (reflecting the evaluation scale from 1 to 9 under different orders) is called the randomness index (R.I.). The ratio of the C.I. value to the R.I. value under the matrix of the same order is called the consistency ratio (C.R.; Larson and Ernest, 2007; Zhu, 2020; Chen et al., 2021b).

Step 7. Calculate the dominance value of each factor.

## Research Methods

This study concludes that when digital content is imported into AI collaborative teaching, teachers choose decision-making factors of AI technology, and then use the AHP hierarchical structure analysis method to establish the dimensions and evaluation criteria that affect teachers’ choice of AI technology. After the overall structure is complete, the expert questionnaire is designed. Through the questionnaire survey, this study has in-depth understanding of the importance ranking and obtained research results, which can be used as a reference for future digital content development and individual recommendation service directions. This first explains the research structure, the content of the expert questionnaire designed by the dimensions and evaluation criteria, then states the sampling and research tools of the research objects and their implementation methods, and finally explains the data processing and analysis of the questionnaire (Liu et al., 2014; Kandlhofer et al., 2016; Huang et al., 2019b).

**Figure 1:**
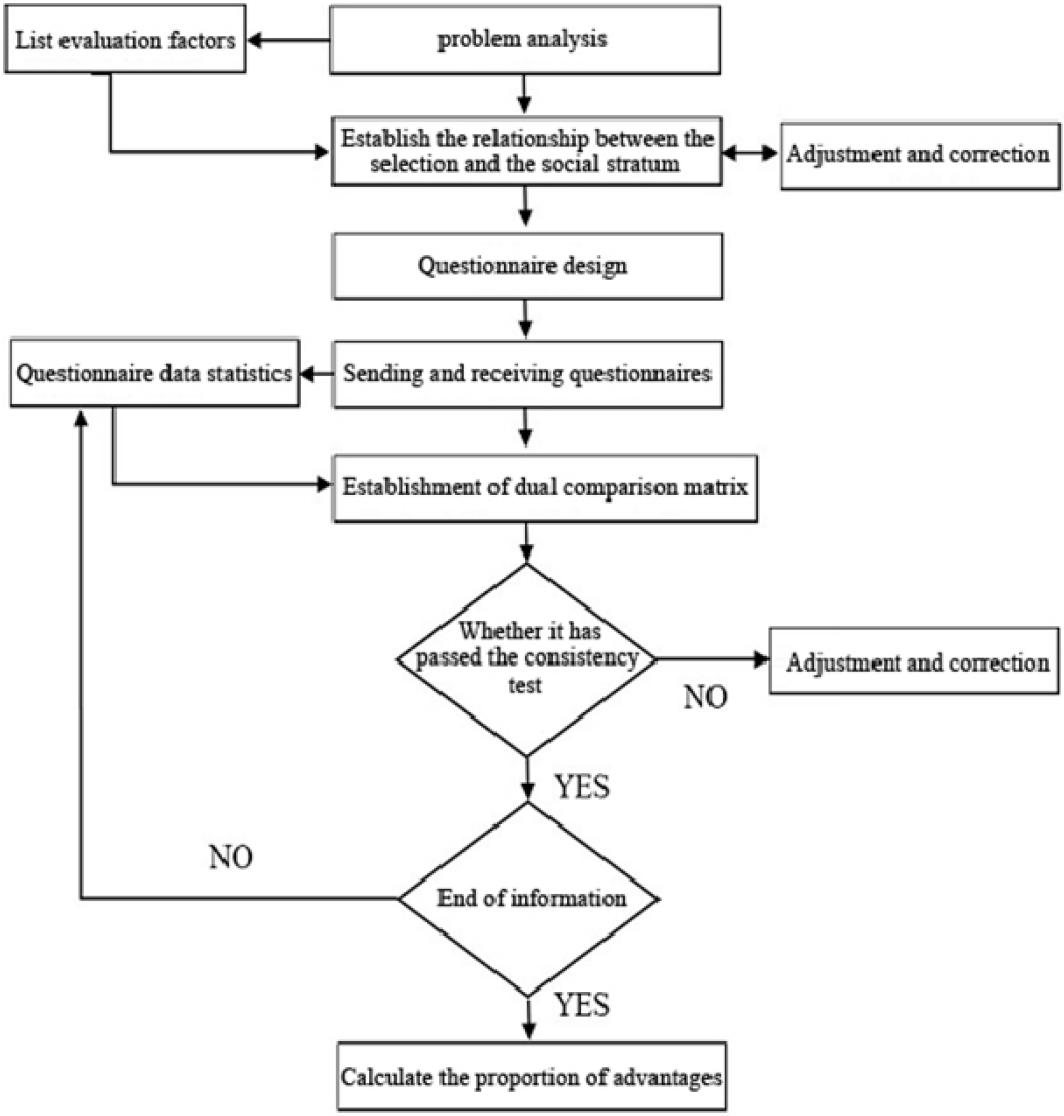
Operation process of AHP method.

When AHP deals with complex issues such as multi-objective or multicriteria for decision-making evaluation, it mainly includes five steps, as shown in Figure 1 (Salavati et al., 2016; Huang et al., 2017; Chen et al., 2019b).

### Research Structure

#### Factors Affecting Teachers’ Choice of AI Technology When Constructing Digital Content and Introducing Artificial Intelligence Into Collaborative Teaching

This study uses the digital content industry to introduce the current status of education intelligence, and then compares the development and application of education intelligence technology and human-computer collaborative teaching methods around the world, and further analyses the model, function, and role of teachers’ emphasis on AI technology, and selects AI for teachers (Huang et al., 2012; Chen et al., 2015, 2020). Decision-making factors relating to science and technology were divided into three dimensions: “modeling characteristics,” “functional characteristics,” and “collaborative tasks.” Twelve evaluation criteria were extrapolated from these three dimensions, as the content options of the research questionnaire (Huang et al., 2004). The breakdown is as follows.

##### Modeling Features

Modeling features refer to the appearance, shape, material, and size of the AI technology, which evoke slightly different psychological feelings in people and influence them in different ways, according to users’ preferences, habits, and level of acceptance (Mayer, 2014; Paas and Sweller, 2014).

##### Features

These refer to AI technology that can learn from massive amounts of data through the system and then correctly interpret external data, using this knowledge to achieve specific goals and complete tasks, including the following: interactive entertainment, record checking, original equipment manufacturer (OEM) maintenance, and reception scheduling (Huang et al., 2006, 2014; Liu et al., 2014).

##### Collaborative Tasks

It can be explored from the four dimensions of “class management,” “teaching observation,” “learning assistance,” and “administrative sharing,” and an example of the current application of AI education technology used in school is shown in Table 1.

**Table 1:** Collaborative tasks of AI technology used in school.

	Zenbo Zenbo Junior	Pepper	Kebbi Air
Class management	Attend the roll call, give medicine to remind smart DJs, interactive games with storytellers, and notify parents	Interactive games, storytelling remote video, simple health education	Interactive games, storytellers, body singing and jumping, pet mode smart assistant, video call
Teaching observation	Remote video, monitoring progress, taking photos and recording, face recognition	Remote video, face recognition	Camera, face recognition video call, pronunciation recognition
Learning aid	Self-learning aids STEAM programming education	Assistant teaching, class assistant accompany STEAM programming education	Knowledge answering questions, object recognition language sense training, conversation practice, STEAM programming education
Administrate sharing	Face recognition, preservation, and help	Face recognition, abnormal warning care detection, temperature sensing and environmental monitoring	Face recognition, remote control of video calls

#### Designing the Content of the Questionnaire

Data collection for this research was divided into two parts. The first was background information on the elementary school teachers, and the second was digital content imported into artificial intelligence and collaborative teaching: factors affecting elementary school teachers’ decision-making when choosing AI technology (Yeung et al., 1998; Wang et al., 2013) (Appendix 1 and Appendix 2).

##### Modeling Characteristics Which Are Inevitable and Cannot Be Avoided

These were as follows: item a: “pet healing,” type a, item b: “animation imagination,” type b, item c: “human simulation,” type c, item d: “natural and invisible,” type d: “collaborative teaching,” B1: interactive entertainment, B2: record check, B3: OEM security, B4: reception schedule, and C: collaborative tasks (C1: class management, C2: teaching observation, C3: learning assistance, and C4: administrative sharing). The scope of this research was limited to all the teachers in a national elementary school. Fifteen people provided feedback (*via* questionnaire surveys) on the factors affecting their decision-making in relation to the selection of AI technology.

#### Research Objects

The scope of this research is limited to all teachers of a national elementary school. Teachers refers to those who taught at the school during the school year, excluding staff, officers, and nurses. The research objects include full-time teachers, acting teachers, directors, and principals. Fifteen people reported their decision-making factors for AI technology selection through questionnaire surveys, as shown in Table 2.

**Table 2:** Respondents and number of questionnaires.

Types of teaching	Positions	Number of teachers	Number of testers	Recovery rate
Grade teacher	Part-time team leader	2	2	
	Concurrent administrative business	4	4	
Subject teacher	Part-time director	2	2	
	Concurrent administrative business	3	3	
	Hourly acting teacher	1	1	
	Special education tour teacher	1	0	
Full-time	Administration principal	1	1	
Total		16	15	93.75%

### Research Tools and Implementation

The questionnaire was divided into two parts: firstly, instructions for filling in the questionnaires and secondly, the questionnaire content, as follows:

Instructions for completing the questionnaire: This mainly entailed explaining the research purpose, research methods, and questionnaire structure so that the teachers filling in the questionnaire would understand the research direction and implementation steps.

**Figure 2:**
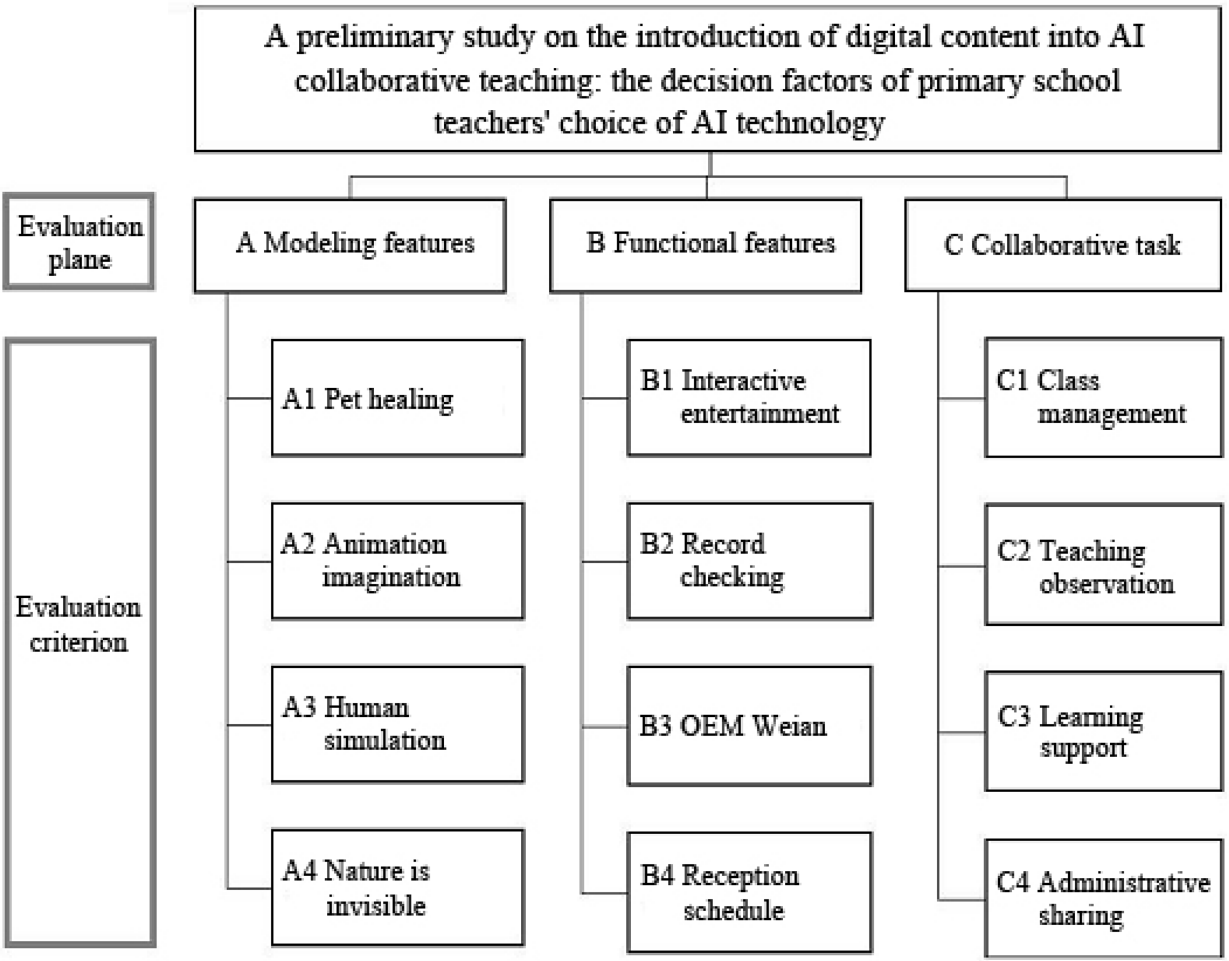
Research hierarchical structure.

Questionnaire content: The main structure was divided into three dimensions: A (“modeling features”), B (“functional features”), and C (“collaborative tasks”), with 12 key factor evaluation criteria. The construction model is shown in Figure 2.

### Data Processing and Analysis

As for the calculation of the maximum eigenvalue, Saaty proposes four approximation methods, among which, the more accurate result can be obtained by using the normalization method of the row vector average value.


$$w_{i}=\frac{1}{n}\overset{n}{\underset{j}{\sum }}\frac{a_{ij}}{\overset{n}{\underset{i=1}{\sum }}a_{ij}}i,j=1,2,\ldots ,n$$ (1)

#### Consistency Verification

Based on the basic assumptions of this theory, assume that A is a consistent matrix, but due to the subjective judgment of the person who fills in the paper, the matrix A may not be consistent, but the evaluation result must pass the consistency test before it can be shown. It is a questionnaire that is consistent with the judgment of the person filling in the questionnaire, otherwise it will be regarded as invalid. Therefore, Saaty suggests using the consistency index (C.I.) and the consistency ratio (C.R.) to verify the consistency of the paired comparison matrix.

#### Consistency Index

The consistency index obtained by the eigenvector method and the difference between *n* (matrix dimension) and the two can be used as a benchmark for judging the degree of consistency.


$$C.I.=\frac{\lambda_{\max}-n}{n-1}$$ (2)

When C.I.=0, it means that the before and after judgments are completely consistent, and C.I>0 means that the before and after judgments are inconsistent. Saaty believes that C.I. ≦ 0.1 is an allowable error.

#### Consistency Ratio

According to the research conducted by Oak Ridge National Laboratory & Wharton School, the consistency index generated from the up-and-down value matrix generated from the evaluation scale 1–9 under different orders is called the randomness index (R.I.), as shown in Table 3.

**Table 3:** Stochastic index table.

Order	1	2	3	4	5	6	7	8	9
R.I.	0.00	0.00	0.58	0.90	1.12	1.24	1.32	1.41	1.45

The ratio of the C.I. value to the R.I. value under the matrix of the same order is called the consistency ratio (C.R.), which is:


$$C.R.=\frac{C.I.}{R.I.}$$ (3)

If C.R. ≦ 0.1, the consistency of the matrix is satisfactory.

The main purpose of the hierarchical analysis method is to quantify qualitative analysis of people’s subjective judgements and finally present the differences between various schemes, with numerical values, to provide decision-makers with a reference. When processing and analyzing questionnaire data, in order to minimize the effects of respondents’ subjective judgements, resulting in inconsistent pairwise comparisons, Saaty recommends the use of a consistency index (C.I.) and consistency ratio (C.R.) and to check the consistency of the dual comparison matrix. The results of the evaluation must pass the consistency test to show that the judgement of the subjects is consistent and not subjectively affected. Screening confirmed that all the questionnaires returned in this study were valid, and the C.I. and C.R. were verified (C.I.<0.1; C.R.<0.1). The scores for the completed questionnaires were quantified using an Excel spreadsheet to calculate the relative weights between each aspect and each evaluation criterion.

## Data Analysis and Discussion

A total of 16 questionnaires was sent out, and all returned questionnaires were valid. Finally, this study carried out a statistical analysis of the survey data and discussed the results.

### Sample Description

Five questionnaires were received from male teachers, and 10 came from female teachers. The age distribution included those who were 41 or above (9, 60%), followed by those who were between 31 and 40years old (5, 33.33%). For education level, the proportion of participants with a master’s degree was the highest (9, 60%), followed by those with one university degree (6, 40%), and those without a college or doctoral degree. Participants’ teaching experience included those with 21 to 30years’ experience (9, 60%), followed by those with 6 to 10years’ experience (4, 26.66%). In terms of teaching category, grade-level teachers concurrently performed administrative services (6, 40%), followed by subject teachers who concurrently performed administrative services (5, 33.33%). Among the teaching grades, the number of senior teachers was the largest (19, 40.43%), followed by the least number of middle-grade teachers (15, 32.91%). The most common teaching field was comprehensive (7, 16.28%), followed by those teaching Mandarin, mathematics, art, and literature (6, 13.95%).

### Verification and Analysis of Questionnaires

In this study, the data collected from the questionnaires were entered into the AHP Excel spreadsheet. The consistency check of C.I. and C.R. values for various dimensions and the evaluation criteria calculation results were less than 0.1, indicating that the judgement of each person filling in the questionnaire was consistent, and the consistency was confirmed. The verification showed that the results of the questionnaire in this study were in good agreement.

### Results Pertaining to Decision-Making Factors Influencing Teachers’ Selection of AI Technology

#### Analysis of the Results of the Questionnaire Survey

##### Analysis of the Relative Weight of the Main Facets

The evaluation facets of this study could be divided into three categories: “modeling features,” “functional characteristics,” and “collaborative tasks.” The weight analysis results for these dimensions were compared with one another. The order of the weight values was found to be as follows: first: collaborative tasks (0.481); second: “functional features” (0.393); and third: “modeling features” (0.126).

The analysis results showed that when digital content is imported into artificial intelligence-based collaborative teaching, “collaborative tasks” capability is the main determining factor in teachers’ decisions about AI technology. Therefore, digital content development should aim to strengthen the planning of collaborative tasks, and time-consuming tasks enable AI robots to relieve teachers of some of their burden, e.g., sharing tedious, repetitive, or time-consuming tasks, enabling them to work together and maximize their full teaching potential. The second most important factor is the “functional characteristics” aspect. Teachers hope to use knowledge to achieve specific goals and tasks through human-computer collaboration and the big data analysis mode. It is also important that the application results can be used as a reference for the development of digital content. As for the “shape feature” aspect, because AI technology is a new form of technology, it is attractive no matter what, but it is not the key to teachers’ consideration.

##### Analysis of the Relative Weight of “Modeling Features” Evaluation Criteria

After analyzing their respective weights through Excel trial calculations, the four evaluation criteria under “sculpting features” were ranked according to relative importance. The first was “A1: pet healing,” with a weight of 0.296; the second was “A2: animation imagination,” with a weight of 0.252; the third was “A4: natural invisible,” with a weight of 0.248; and the fourth was “A3: human simulation,” with a weight of 0.203.

##### Analysis of the Relative Weight of “Functional Characteristics” Evaluation Criteria

After the AHP trial calculations and after comparing their weights with one another, the four evaluation criteria under “functional features” were ranked according to relative importance. The first was “B3: OEM security,” with a weight of 0.327, and the second was “B2: record inspection.” The weight of “core” was 0.262; the weight of the third criterion, “B1: interactive entertainment,” was 0.224; and the weight of the fourth criterion, “B3: reception schedule,” was 0.188.

##### Analysis of the Relative Weights of the Evaluation Criteria for “Collaborative Tasks”

After analyzing the respective weights of each criterion in Excel calculations, the four evaluation criteria under “collaborative tasks” were ranked according to relative importance. The first was “C3: learning assistance,” with a weight of 0.382; the second was “C2: teaching observation,” with a weight of 0.260; the third was “C4: administrative sharing,” with a weight of 0.185; and the fourth was “C1: class management,” with a weight of 0.172.

#### Overall Ranking of Decision-Making Factors Affecting Teachers’ Choices of AI Technology

On the basis of the weight data relating to various aspects of decision-making and the evaluation criteria in this research, the overall weight was calculated. The overall weight ranking and the overall weight cumulative table are shown in Table 4. The ranking of the weighted data in the table clearly indicates how important various factors are to elementary school teachers when it comes to choosing AI technology and when digital content is to be imported into artificial intelligence-based collaborative teaching.

**Table 4:** Overall weight analysis table and radar chart of factors affecting decisions about choice of AI technology by elementary school teachers.

Facet	Facet weight	Facet ranking	Evaluation criteria	Evaluation criteria local weight	Evaluation criteria overall weight	Evaluation criteria overall ordering
Modeling features	0.126	3	A1 Pet Healing	0.2963	0.0374	9
			A2 Animation Imagination	0.2525	0.0318	10
			A3 Human simulation	0.2035	0.0257	12
			A4 Naturally invisible	0.2478	0.0312	11
Functional characteristics	0.393	2	B1 Interactive Entertainment	0.2239	0.0880	6
			B2 Record check	0.2629	0.1030	4
			B3 Security	0.3267	0.1284	2
			B4 Reception schedule	0.1876	0.0737	8
Collaborative tasks	0.481	1	C1 Class management	0.1722	0.0828	7
			C2 Teaching observation	0.2600	0.1250	3
			C3 Learning support	0.3825	0.1840	1
			C4 Administrate sharing	0.1854	0.0892	5

The results of the study indicate that in the ranking of overall weight values of the 12 evaluation criteria, the top four (with the highest weights) were “C3: learning assistance” (0.1840) and “C2: teaching observation” (0.1250) in category C (collaborative tasks); and “B3: OEM maintenance” (0.1284) and “B2: record check” (0.1030) in category B (functional features).

#### Discussion

The results show that teachers believe that the most important factor in decision-making for AI technology is the planning of collaborative tasks, enabling them to implement differentiated teaching and level the learning gap for students. Secondly, the service demand for teaching observation has increased significantly with educational reform. AI technology uses sensor technology to assist teachers in recording the actual situation in the classroom, which is convenient for teachers when they want to focus on discussion, analysis, evaluation, and feedback; whether it is for the purposes of evaluating teachers’ teaching methods or students’ learning strategies, it helps to have another pair of “friendly eyes” to help professional diligence. In addition, in terms of the functional characteristics of AI technology, teachers need AI technology to be able to share the work of supervisors inside the classroom and off campus (including taking account of traffic and maintaining a tidy campus) so that teachers have more time to take care of their students at the class level or can safely leave the classroom temporarily to deal with other administrative duties. However, the development of human-computer collaboration is still at an intermediate stage. AI technology can become a human teaching partner or an administrative assistant, and it can divide labor and cooperate with other systems to improve students’ learning while teachers maintain responsibility for planning, communication, coordination, and decision-making (Kandlhofer et al., 2016). AI technology can support individualized teaching and guidance. In the future, AI technology will gain a dominant position as an elite teaching consultant; even after reaching the super stage, humans and AI technology will be integrated, and human senses will be expanded. By then, the potential for human-computer action and development will only be limited by human beings’ thinking and imagination.

#### Comparison of Collaborative Teaching Versus Lecturing

**Figure 3:**
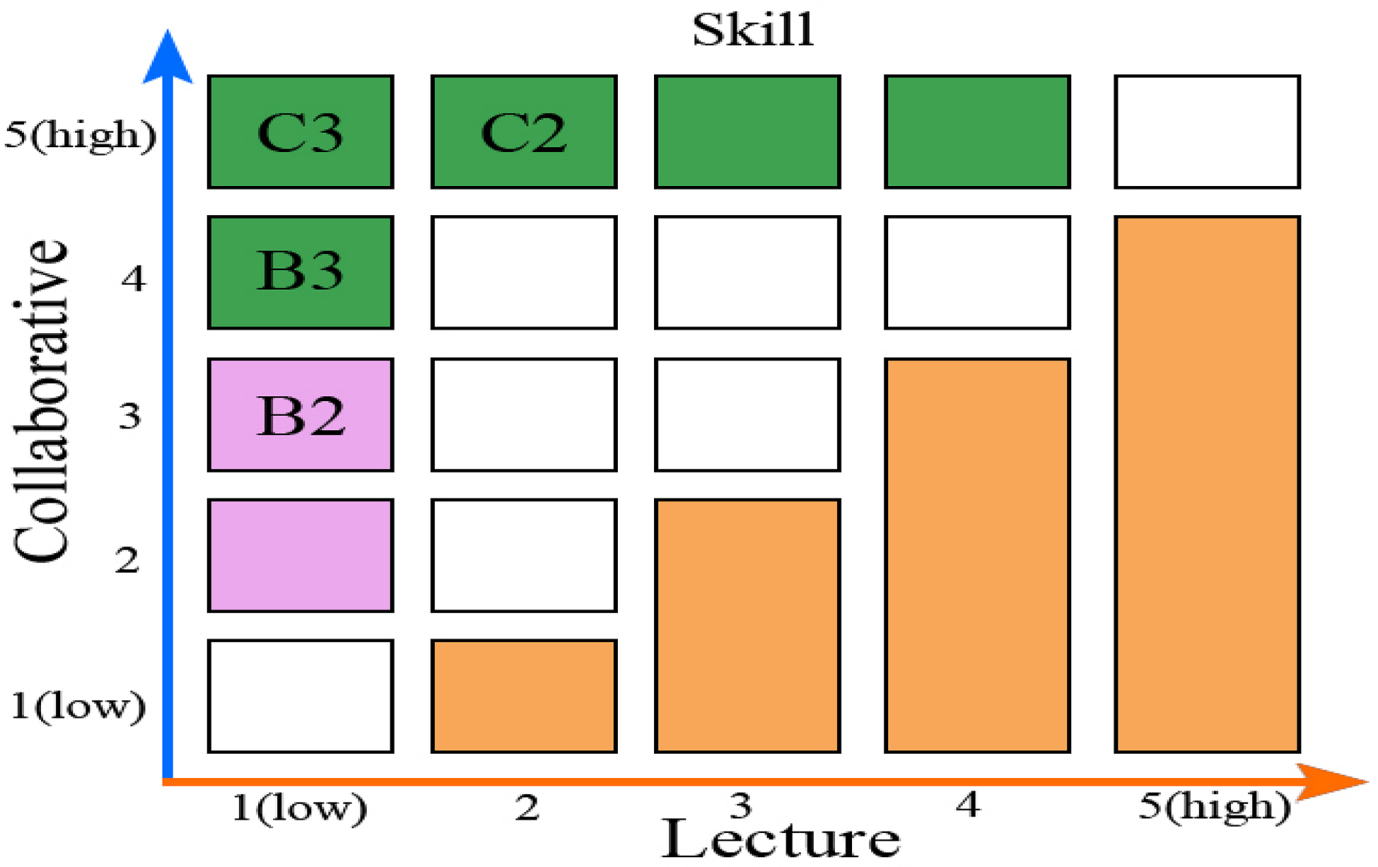
Comparison of collaborative teaching versus lecturing.

For comparisons among the criteria, to evaluate the behavior of each alternative against the factors, a Likert-type scale of 1 to 5 was used, in which a higher value indicates better behavior regarding the evaluated factor, as shown in Figure 3. The value obtained for the consistency index (C.I.) which indicates an acceptable level of consistency. The relationships between the skills and the different criteria and compared collaborative teaching with traditional lectures are shown in Figure 3. Improvement is seen in the acquisition of skills by applying the collaborative teaching methodology versus lecturing. Each of the skills is linked with several factors, and the same factor influences various skills. The valuation of each skill is represented in an X-Y diagram, where the X value represents the average value obtained for the lectures, which is calculated using the assessment average of the factors that influence the skill. Similarly, the Y value represents the average value obtained for the collaborative methodology. All values indicate that the collaborative teaching methodology is better for the acquisition of skills than lectures. The most important improvement occurs for skills “C3: learning assistance” and “C2: teaching observation”; and “B3: OEM maintenance” and “B2: record check”.

## Conclusion

When digital content is imported into artificial intelligence-based collaborative teaching, key factors are taken into consideration when teachers choose AI technology. Through the perspective of elementary school teachers, this study has gained a better understanding of the real needs of teachers in artificial intelligence-based collaborative teaching, and the significance of both the convenience and inherent limitations of smart technology. The research results’ contribution can be used as a reference to help determine the direction of digital content development and individual recommendation services. The most important aspect of this research evaluation of the data was sorting by factors, followed by “collaborative tasks,” “functional features,” and “modeling features,” that elementary school teachers are most concerned about with regard to AI technology. The provided collaborative tasks are the most helpful and present the least psychological burden for them when digital content is imported into artificial intelligence-based collaborative teaching. Therefore, future digital content development strategies should endeavor to strengthen the design and planning of collaborative tasks in educational settings that use AI technology, which is recommended for teachers seeking to individualize their services and differentiate module design according to different teaching fields and categories.

The results of this research evaluation of the data were sorted by weight, the ranking of weights with regard to the seventh and eighth evaluation factors is worth exploring further, namely “class management” and “reception ranking.” “Class management” (8.28%) is an indispensable teaching art for elementary school teachers, which comprehensively displays the following: the teacher’s personal educational philosophy, time management methods, life order norms, reward systems, competition guidance skills, communication skills, crisis management skills, counselling skills, and activity leadership methods, etc. In addition, “reception ranking” (7.37%) is regarded as a part of parent-teacher communication and time management. Teachers’ working days are invariably full and intensive. If they cannot schedule their workflow quickly and effectively, they may miss the most suitable time for incident handling, thereby creating further work and trouble for themselves (e.g., difficulties associated with timing, channels, tools, frequency, and terms of communication between parents and teachers). However, some subject teachers hope that AI technology can assist in class management, activity leadership, rewards, and punishments, and enable them to work more efficiently so that they can focus purely on teaching. The greater the capacity for human simulation with the AI technology in question, the more favorable it is perceived as being, but a research limitation exists: if the AI technology exceeds a certain critical point, favorability will decrease. Elementary school teachers in the lower grades believe that human-simulated robots will upset or frighten younger students, which is not conducive to collaborative teaching. In future work, this study can further discuss teaching innovations in digital media education, aimed at improving the quality and effectiveness of teaching and learning.

## Data Availability Statement

The original contributions presented in the study are included in the article/supplementary files, further inquiries can be directed to the corresponding author.

## Author Contributions

All authors contributed to the study conception and design. Material preparation, data collection, and analysis re-performed by ZL. The first draft of the manuscript was written by WW.

## Conflict of Interest

The authors declare that the research was conducted in the absence of any commercial or financial relationships that could be construed as a potential conflict of interest.

## Publisher’s Note

All claims expressed in this article are solely those of the authors and do not necessarily represent those of their affiliated organizations, or those of the publisher, the editors and the reviewers. Any product that may be evaluated in this article, or claim that may be made by its manufacturer, is not guaranteed or endorsed by the publisher.

## Appendix

**Questionnaire Content**

**1. Survey on the importance of each evaluation dimension.**

**Table:**

Evaluation dimension	Description of content
A. Modeling features	Refer to the appearance, shape, material, and size of AI technology. The psychological feelings and influence it brings to people are slightly different. They are different depending on the user’s own preferences, habits, and acceptance. According to the current market we can see that how AI technology is received can be divided into: pet healing type, animation imagination type, human simulation type, and self. However these are intangible types
B. Functional characteristics	Refers to AI technology that can learn from massive amounts of data through the system, and then correctly interpret external data, and use this knowledge to achieve specific goals and tasks, including: interactive entertainment, record checking, OEM maintenance, and reception arrangements
C. Collaborative tasks	Refers to what role AI technology plays in the teaching scene, and what tedious, repetitive, or dangerous tasks are shared for teachers. How to assist teachers to exert the power of one plus one greater than one in the process of collaborative teaching? From the above thinking points, you can start from “class management,” “teaching observation,” and the four dimensions of “learning assistance” and “administrative sharing”

Rank the importance of the above evaluation dimensions: ______≥_______≥______ (please fill in the code)

**2. Analysis of the importance of each evaluation facet.**

**Table:**

Evaluation dimension	The relative importance of evaluation dimensions									
	Absolutely important	Extremely important	Quite important	Slightly important	Equally important	Slightly important	Quite important	Extremely important	Absolutely important
	1:9	7:1	5:1	3:1	1:1	1:3	1:5	1:7	1:9
A. Modeling features									
B. Functional features									
C. Cooperative tasks									

(Please compare the relative importance of each aspect according to the above sorting, please tick).

**Sub-dimension analysis questionnaire-evaluation criteria weight table.**

**Table:**

Evaluation dimension	Facet weight	Critical factor evaluation criteria	Evaluation criteria weight
**A. Modeling features**	0.126	A1 Pet healing	0.296
		A2 Animation Imagination	0.252
		A3 Human simulation	0.203
		A4 Naturally invisible	0.248
**B. Functional features**	0.393	B1 Interactive entertainment	0.224
		B2 Record check	0.262
		B3 OEM Wei’an	0.327
		B4 Reception schedule	0.188
**C. Collaborative task**	0.481	C1 Class management	0.172
		C2 Teaching observation	0.260
		C3 Learning support	0.382
		C4 Administrative sharing	0.185
